# Possible Role of Docosahexaenoic Acid in Response to Diarrhetic Shellfish Toxins in the Mussel *Perna viridis*

**DOI:** 10.3390/md21030155

**Published:** 2023-02-25

**Authors:** Kuan-Kuan Yuan, Zi-Min Chen, Ya-Xin Liu, Hong-Ye Li, Wei-Dong Yang

**Affiliations:** Key Laboratory of Aquatic Eutrophication and Control of Harmful Algal Blooms of Guangdong Higher Education Institute, College of Life Science and Technology, Jinan University, Guangzhou 510632, China

**Keywords:** diarrhetic shellfish toxins, DHA, esterification, Nrf2 signaling pathway, qPCR, histopathology

## Abstract

Marine bivalves are rich in docosahexaenoic acid (DHA), a polyunsaturated fatty acid known to be beneficial for human health; however, the potential role of DHA in protecting shellfish from the toxicity of diarrhetic shellfish toxins (DSTs) remains poorly understood. Here, we aimed to study the effect of DHA on the response of the bivalve, *Perna viridis*, to DSTs by using LC-MS/MS, RT-qPCR, and histological examination. In this study, we observed that the DHA content decreased significantly with esterification of DSTs in the digestive gland of the mussel *P. viridis* after 96 h of exposure to *Prorocentrum lima*, a DST-producing dinoflagellate. The addition of DHA significantly increased the esterification level of DSTs and increased the expression of Nrf2 signaling pathway-related genes and enzyme activities, alleviating the damage of DSTs to digestive glands. These results suggested that DHA may mediate the esterification of DSTs and activation of the Nrf2 signaling pathway in *P. viridis* to protect mussels from the toxic effects of DSTs. This study may provide new insights regarding the response of bivalves to DSTs and lay the foundation for uncovering the role of DHA in environmental adaptation of bivalves.

## 1. Introduction

Among the widely distributed marine biotoxins, diarrhetic shellfish toxins (DSTs) are commonly associated with diarrhetic shellfish poisoning events worldwide [1,2,3]. DSTs belong to a group of polyether lipid-soluble compounds, which mainly include okadaic acid (OA) and dinophysistoxin (DTX) analogues [4,5]. They are produced by some marine dinoflagellate species of the genera *Prorocentrum* and *Dinophysis* [6]. As specific inhibitors of serine/threonine protein phosphatases, these toxins can cause severe mucosal damage in the intestine, disrupt DNA structure and cytoskeleton, and exert negative immunological and neurological effects [7,8,9]. The predominant route of human exposure to DSTs is via the consumption of contaminated filter-feeding shellfish, which can lead to gastrointestinal disturbance and diarrhetic poisoning [10,11]. Studies have shown that compared to other organisms, bivalves exhibit tolerance to DSTs to a certain extent, and the toxicity of DSTs to bivalves decreases with exposure time [12,13,14,15].

In bivalve mollusks, the digestive gland is the main organ that accumulates DSTs, which are transformed during digestion [16]. Studies have shown that free forms of DSTs (OA, DTX1, DTX2) produced by marine microalgae can be esterified with fatty acids of different carbon chain lengths to form 7-O-acyl derivatives, known as dinophysistoxin-3 (DTX3) [17,18,19]. DTX3 is less toxic due to its low affinity for the target protein [8], which may also contribute to the tolerance of bivalves to DSTs.

Studies have demonstrated that DSTs can trigger oxidative stress in bivalve shellfish [13,14,15]. Nuclear factor E2-related factor (Nrf2) is as an essential transcription factor that prevents exogenously-induced oxidative damage by regulating the expression of antioxidant proteins [20]. Several studies have suggested that the target genes of the Nrf2/ARE signaling pathway, such as glutathione-S-transferases (GSTs), ABC transporters, and glutathione peroxidase (GPx), and glutathione reductase (GR), are involved in metabolic detoxification of DSTs in bivalves [12,21,22,23,24,25]. Nevertheless, the regulatory mechanism associated with the Nrf2/ARE signaling pathway in bivalves after exposure to DSTs remains largely unclear.

Marine bivalves are rich in omega-3 long chain polyunsaturated fatty acids (LC-PUFA), and their diet mostly contains marine phytoplankton species, which are the main producers of omega-3 LC-PUFA [26]. Although many invertebrates can produce omega-3 LC-PUFA, bivalves generally have limited ability to do so [27]. Docosahexaenoic acid (DHA) is a major omega-3 LC-PUFA with antioxidant activity, which is mainly because of its high level of unsaturation. It can be easily per-oxidized to form J_3_-isoprostane, and it directly reacts with Keap1, a negative regulator of Nrf2, initiating the dissociation of Keap1 from Cullin3, thereby activating the Nrf2/ARE signaling pathway [28]. However, the role of DHA in bivalves remains unclear. So far, only a few studies have reported that supplementation of diet with DHA improves the daily growth rate and survival of bivalves [29,30,31]. To the best of our knowledge, it is not known whether DHA contributes to tolerance of bivalves to DSTs. 

The mussel *Perna viridis* has been used in toxicological investigations due to its tolerance to environmental changes [32,33]. The dinoflagellate *P. lima*, a common DST-producing algae, has been widely used in DST-related toxicology research [34]. To investigate the potential role of DHA in protecting shellfish from the harmful effects of DSTs, we assessed the effect of DHA on the accumulation and esterification of DSTs, and observed the changes in metabolic detoxification, antioxidant gene expression, and damage to digestive gland tissues in the *P. lima*-exposed *P. viridis* after the addition of DHA. This study will improve our understanding regarding the role of DHA in inducing tolerance to DSTs in bivalve mollusks.

## 2. Results

### 2.1. Changes in Accumulation and Esterification of DTSs after Exposure to P. lima

**Figure 1:**
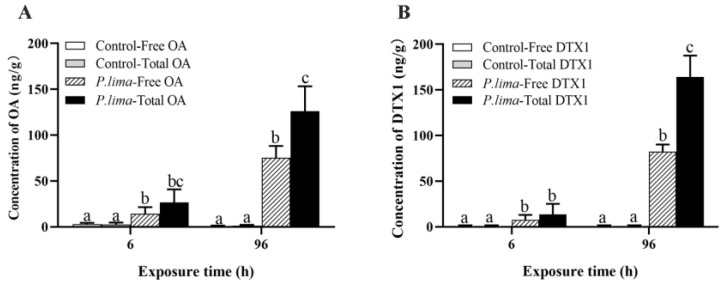
Accumulation of OA and DTX1 in the digestive gland of the mussel *P. viridis* after exposure to *P. lima* (in ng/g wet weight). (**A**) OA. (**B**) DTX1. Control, fed with *T. subcordiformis* (1 × 10^7^ cells/L); *P. lima*, fed with *T. subcordiformis* (1 × 10^7^ cells/L) and *P. lima* (2 × 10^6^ cells/L). Data are presented as mean ± SD (*n* = 3). Different letters indicate significant differences (Duncan’s *t*-test, *p* < 0.05).

During the experiment, the mussels in each group grew well without mortality, indicating that *P. viridis* was highly tolerant to DSTs. In the *P. lima*-exposed group, the levels of total OA (OA+OA ester) and DTX1 (DXT1+DXT1 ester) were slightly higher than those of the free forms of OA and DTX1 after 6 h of exposure, although the difference was not statistically significant. However, the levels of total OA and DTX1 were significantly higher than those of free OA and DTX1 (*p* < 0.05) at 96 h (Figure 1), indicating that DSTs underwent esterification in *P. viridis*, which increased with exposure time.

### 2.2. Changes in Fatty Acid Levels in the Digestive Gland after Exposure to P. lima

**Figure 2:**
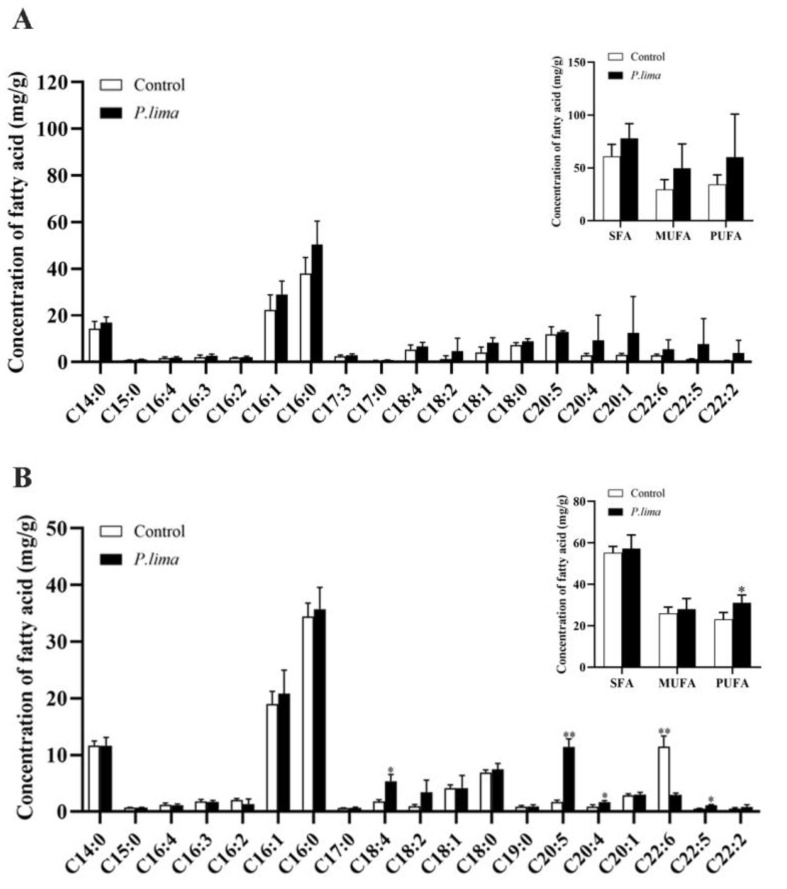
Fatty acid concentration (in mg/g dry weight) in the digestive gland of the *P. lima*-exposed mussels at 6 h (**A**) and 96 h (**B**). SFA, MUFA, and PUFA represent saturated fatty acids, monounsaturated fatty acids, and polyunsaturated fatty acids, respectively. Data are presented as mean ± SD (*n* = 3). Significant differences compared to control are represented by asterisks (*t*-test, * *p* < 0.05. **p < 0.01).

As shown in Figure 2, the levels of fatty acids in the digestive gland did not change significantly compared to that in the control group after 6 h of exposure to *P. lima* (Figure 2A). However, the levels of some PUFA, including eicosapentaenoic acid (EPA) and DHA, were significantly altered after 96 h. The level of EPA C20:5 (n−3) increased significantly, while DHA content decreased sharply (from 11.5 to 3 mg/g) (*p* < 0.05) (Figure 2B). The DHA content correlated with the extent of DST esterification, suggesting that DHA might be involved in the esterification of DSTs.

### 2.3. Changes in Nrf2/ARE Signaling Pathway in the Digestive Gland after Exposure to P. lima

**Figure 3:**
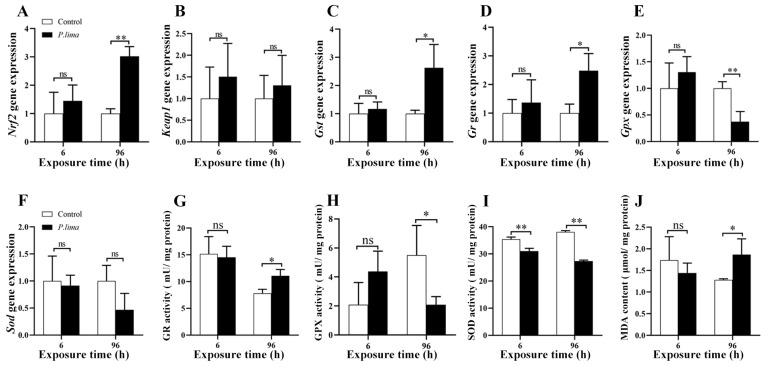
Changes in gene expression, antioxidant enzyme activity and MDA level in the digestive gland of the mussel *P. viridis* after exposure to *P. lima.* (**A**) *Nrf2* expression. (**B**) *Keap1* expression. (**C**) *Gst* expression. (**D**) *Gr* expression. (**E**) *Gpx* expression. (**F**) *Sod* expression. (**G**) GR activity. (**H**) GPx activity. (**I**) SOD activity. (**J**) MDA level. Control, fed with *T. subcordiformis* (1 × 10^7^ cells/L); *P. lima*, fed with *T. subcordiformis* (1 × 10^7^ cells/L) and *P. lima* (2 × 10^6^ cells/L); Data are presented as mean ± SD (*n* = 3). Significant differences compared to control are represented by asterisks (*t*-test, * *p* < 0.05, ** *p* < 0.01, ns represents non-significance).

As shown in Figure 3, *Nrf2* was significantly upregulated (*p* < 0.01), as was the expression of its downstream genes, *Gst* and *Gr* (*p* < 0.05), while the level of the *Gpx* transcript decreased (*p* < 0.01) after 96 h of exposure to *P. lima*. *Sod* expression did not change significantly. After exposure to *P. lima*, the activity of GR increased at 96 h *(p* < 0.05), GPx activity decreased at 96 h *(p* < 0.05), whereas SOD activity *(p* < 0.01) decreased at 6 h and 96 h, and MDA level increased significantly (*p* < 0.05) at 96 h in the digestive gland. These results suggested that *P. lima* exposure induced oxidative stress and activated the Nrf2 signaling pathway in mussels.

### 2.4. Changes in the Esterification Level of DSTs after Addition of DHA

**Figure 4:**
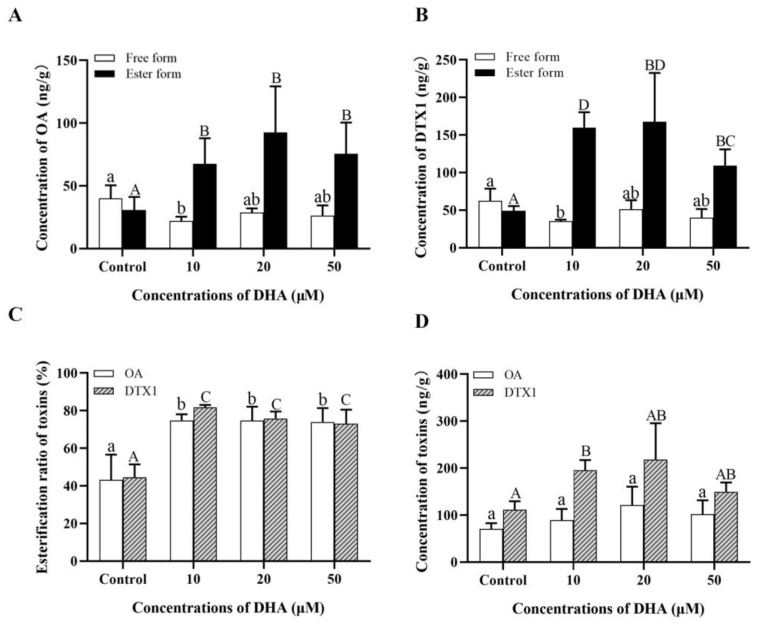
Changes in esterification level of DSTs after addition of DHA. (**A**) Contents of OA. (**B**) Contents of DTX1. (**C**) Esterification ratio of DSTs (%). (**D**) The total content of DSTs. Data are presented as mean ± SD (*n* = 3). Different letters indicate significant differences (Duncan’s *t*-test, *p* < 0.05).

The levels of the free and ester forms of OA and DTX1 in the *P. lima*-exposed mussels after DHA addition are shown in Figure 4A,B, and the esterification ratios of DSTs are shown in Figure 4C. The esterified OA and DTX1 levels and esterification ratios of OA and DTX1 in all DHA-treated groups (10 µM, 20 µM, 50 µM) were significantly higher than those in the control group (0 µM) (*p* < 0.05) after 96 h of exposure to *P. lima*. In particular, the lowest concentration of DHA (10 µM) significantly decreased the accumulation of free OA (from 40.03 to 22.07 ng/g) and DTX1 (from 62.37 to 35.46 ng/g), but increased the accumulation of total DTX1 (Figure 4D).

### 2.5. DHA Reduced Damage Caused by DSTs in the Digestive Gland

**Figure 5:**
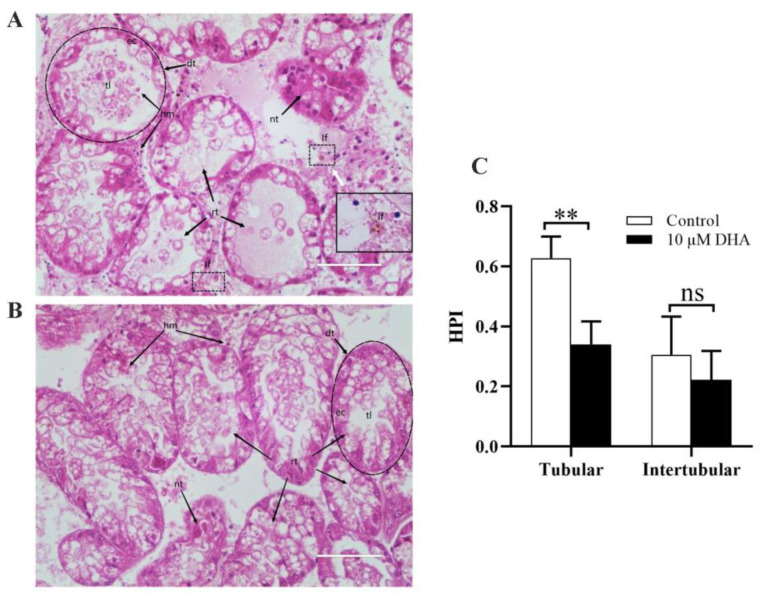
Histological sections of digestive glands of *P. viridis* (HE staining). (**A**) Digestive glands of the *P. lima*-exposed mussels after the addition of DMSO. Digestive tubules (dt) were composed by a single layer of orderly arranged ciliated eosinophilic epithelial cells (ec). Note the thinned epithelial cell (ec) layer and dilated tubular lumen (tl) during the disintegrating phase of the regressing tubule (rt), which is characterized by “shedding” of the epithelial cells into the tubule lumen (tl) and degradation. There was extensive hemocyte infiltration (hm) in the damaged diverticulum, lipofuscin aggregates (If) near the damaged tubules, and necrotic (nt) in some tubules. Scale bars: 50 μm. (**B**) Digestive glands of the *P. lima*-exposed mussels after the addition of DHA (10 µM). Regressing tubules (rt) may be in the reconstituting phase, with widened epithelial cell (ec) layer and narrow or almost occlusive tubule lumen (tl). There was limited hemocyte infiltration (hm) in the damaged diverticulum. Scale bars: 50 μm. (**C**) Histopathological condition indices of the *P. lima*-exposed mussels after the addition of 10 µM DHA. Data are presented as mean ± SD (*n* = 3). Significant differences compared to control are represented by asterisks (*t*-test, **p < 0.01, ns represents non-significance).

The histological alterations in the digestive gland were evaluated using a semi-quantitative histopathological index. After exposure to *P. lima*, the digestive gland of *P. viridis* was severely damaged, mainly characterized by regressing tubule and common dilation of the tubule lumen. This might be attributed to severe atrophy of the epithelium, thinner layers of the epithelial cell, extensive hemocyte infiltration in the damaged tissue, and frequent lipofuscin aggregation around the epithelial cells. Furthermore, some digestive tubules developed deformation or even necrosis (Figure 5A). In the 10 µM DHA group, tubule atrophy was significantly alleviated, as the thickness of the epithelial cells increased significantly, the tubule lumen narrowed and was almost occluded, and hemocyte infiltration was limited to sporadic distribution, although tubular necrosis was still observed (Figure 5B). The HPI per reaction pattern in the digestive gland tissues is shown in Figure 5C. Tubular alterations were the observed reaction pattern with the highest HPI, and the addition of DHA significantly reduced the high indices induced by DSTs. Regarding intertubular morphology, DHA appeared to reduce HPI, although it was not statistically significant.

### 2.6. DHA Activated the Nrf2/ARE Signaling Pathway

**Figure 6:**
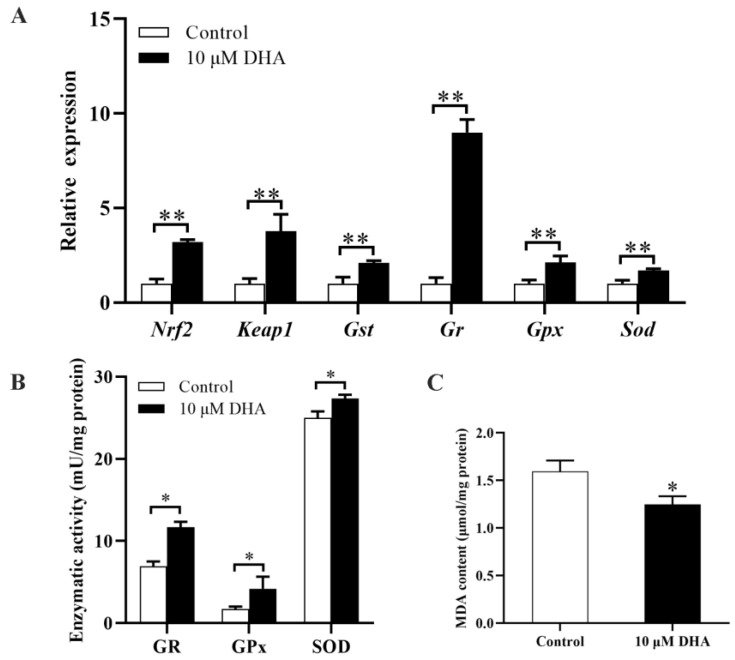
Changes in gene expression, antioxidant enzyme activity and MDA level in the digestive gland of the *P. lima*-exposed mussels after the addition of 10 µM DHA. (**A**) Alterations in expression of Nrf2, Keap1, Gst, Gr, Gpx, and Sod revealed by RT-qPCR. (**B**) Activities of GR, GPx, and SOD. (**C**) MDA level. Data are presented as mean ± SD (*n* = 3). Significant differences compared to control are represented by asterisks (*t*-test, * *p* < 0.05, ** *p* < 0.01).

As shown in Figure 6A, Nrf2 and Keap1 expression was significantly higher in the presence of DHA (10 µM) than in the absence of DHA (*p* < 0.01) after exposure to *P. lima*. Gst (*p* < 0.01), Gr (*p* < 0.01), and Sod (*p* < 0.01) were significantly upregulated, and the activities of GR (*p* < 0.05), GPx (*p* < 0.05), and SOD (*p* < 0.01) increased significantly (Figure 6B), whereas the level of MDA (*p* < 0.05) decreased significantly in the presence of DHA (10 µM) (Figure 6C). These results indicated that DHA may enhance antioxidant capacity by activating the Nrf2 signaling pathway.

## 3. Discussion

Bivalves are filter-feeding mollusks that can accumulate phytotoxins by filtering toxic algae. Our previous studies have demonstrated that the mussel, *P. viridis*, a typical environmental organism, has developed a cytoprotective mechanism to attenuate the detrimental effects of DSTs [21,25,35,36,37]. Recently, several studies have found that DSTs are esterified in bivalves, which can reduce their toxicity and possibly contribute to DST tolerance [17,18,19,38,39]. However, mussels are suggested to have a relatively weak esterification capacity to DSTs compared with other shellfish such as clams [40,41], which obfuscates the role of esterification in the tolerance of mussel to DSTs. It remains unclear whether esterification plays a key role in the tolerance of *P. viridis* to DSTs. In line with our previous results [42], here we also found that the concentration of total DSTs (DSTs + DST ester) was significantly higher than that of free DSTs in the digestive gland of the mussel 96 h after exposure to *P. lima* (Figure 1), indicating the presence of esterified DSTs in the mussel.

DHA is one of the most important omega-3 fatty acids mainly found in seafood, such as fish, shellfish, and fish oils. The marine bivalve lipids are a source of high-quality lipids beneficial for human health and have been considered a sustainable future source of natural omega-3 PUFAs [26,43]. However, although DHA is abundant in marine bivalves, studies on the function of DHA in bivalves are limited [29,44]. Recently, Qiu et al. (2020) found that the fatty acid content in the mussel, *Mytilus galloprovincialis,* decreased progressively with the accumulation and esterification of DSTs, and that the content of PUFA, especially DHA, decreased, suggesting that DHA may be the main fatty acid involved in DST esterification [39]. In the current study, the content of DHA decreased significantly with the esterification of DSTs in the digestive gland of the *P. lima*-exposed mussel. The addition of different concentrations of DHA significantly increased the content of esterified OA and DTX1, and 10 µM DHA even reduced the content of free DSTs. These results suggested that DHA might be one of the fatty acids essential for the esterification of DSTs. The increase in EPA and stearidonic acid (SDA) contents may be due to the higher EPA and SDA content in the *P. lima* strain CCMP 2579 (Figure S2). It may also be that DHA as a substrate participates in esterification of DSTs, resulting in the compensatory synthesis of EPA and SDA as important precursors in the endogenous synthesis of polyunsaturated fatty acids [27,45].

The Nrf2/ARE signaling pathway is a critical mediator of oxidative response and plays an important protective role against oxidative damage [20]. In this study, we observed that the expression of *Nrf2* and its downstream target genes, *Gst* and *Gr*, were upregulated, indicating the activation of the Nrf2 signaling pathway after exposure to *P. lima*. Similar to our previous results [35], *Gpx* was downregulated after exposure to *P. lima*, which appears to contradict the activation of the Nrf2 signaling pathway. Several studies have shown that activation of NR1J nuclear receptor groups (including HR96) may lead to increased expression and activity of antioxidant enzymes such as GPx [46]. However, whether there is any crosstalk between nuclear receptor and the Nrf2 signaling pathway remain unknown. Correspondingly, GR activity increased, but the activities of GPx and SOD significantly decreased and MDA level increased, indicating that the activation of Nrf2 pathway was not sufficient to reverse the oxidative stress caused by DSTs. These are consistent with our previous findings [25,35,37]. However, the underlying molecular mechanism via which DSTs induced the Nrf2 signaling pathway remains unclear. Previously, we have shown that the DSTs activated the JNK signaling pathway, which was a typical PP1/PP2A phosphatase inhibitor; thus, we speculated that it might activate the Nrf2 signaling pathway via phosphorylation. However, owing to the differences in Nrf2 phosphorylation sites between mussels and mammals, we were not able to observe changes in Nrf2 phosphorylation levels using the only commercially available S40 phosphorylation antibody [25]. Therefore, the activation of the Nrf2 signaling pathway cannot be solely attributed to the action of DSTs as phosphatase inhibitors.

DHA has been demonstrated to be per-oxidized to produce J_3_-isoprostane, which can activate the Nrf2 signaling pathway [28]. DHA supplementation in HepG2 culture medium can significantly increase the expression and activity of SOD, CAT, and GPx, and reduce the level of reactive oxygen species [47]. Magalhães et al. (2022) found that dietary DHA could increase the activities of SOD and GR in the liver of gilthead sea bream [48]. To reveal the possible roles of DHA in shellfish tolerance to DSTs, we observed the changes in metabolic detoxification, antioxidant gene expression, and damage to digestive gland tissues in the *P. lima*-exposed *P. viridis* after addition of 10 µM DHA. We found that *Nrf2* and its downstream genes *Gst*, *Gr*, *Gpx*, and *Sod* were significantly upregulated in the 10 µM DHA group compared to that in the *P. lima*-exposed group without DHA, and that the activity of the antioxidant enzyme increased significantly, while MDA level decreased. These results suggested that DHA may be per-oxidized in the digestive gland after exposure to *P. lima*, thereby activating the Nrf2/ARE signaling pathway.

Histopathology of bivalves is a widely recognized tool used in studies on environmental toxicology and the most direct way to visualize the physiological changes in organisms exposed to various toxic substances [49,50]. The histological alterations induced by DSTs mainly manifested as tubule atrophy, hemocyte infiltration, digestive cell reduction, and epithelial cell atrophy, as reported by Neves et al. [51]. Similar morphological changes were observed in the digestive gland tissues of the *P. lima*-exposed *P. viridis*. However, after the addition of 10 µM DHA, the extent of atrophy of the degenerated tubules reduced, as was evident from the widening of the epithelial cell layer, narrowing of the tubule lumen, and reduction in hemocyte infiltration, suggesting that DHA could reduce the damage caused by DSTs to mussels. The recovery of the damage to digestive gland tissues after adding DHA was consistent with the esterification of DSTs and activation of the Nrf2 signaling pathway, suggesting that DHA might reduce the toxicity of DSTs to shellfish by activating the Nrf2 signaling pathway and promoting esterification. However, how DHA activates the Nrf2 signaling pathway and promotes the esterification of DSTs in bivalves, and the fatty acid profile of esterified DSTs in *P. viridis* is still unclear and deserves further study. On the other hand, the present study only observed the response of shellfish exposed to DSTs 96 h after 2 h of DHA addition, which helps to understand the role of DHA in the esterification of DSTs and the mechanism of shellfish tolerance to DSTs. However, it is not clear whether adding DHA after DST exposure can alleviate the damage caused by toxins to shellfish. This is of great significance for the disposal of shellfish after the occurrence of DSTs contamination, which is worthy of further study and implications should be discussed in the broadest context possible. Future research directions may also be highlighted.

## 4. Materials and Methods

### 4.1. Animal Maintenance and Algae

The mussel *P. viridis* was purchased from a local seafood market in Guangzhou, which collected them from Zhanjiang, China. The individual mussels selected were 9.5 ± 1 cm long, the morphology as shown in Figure S1, and their soft body weight was 7.9 ± 1 g; they were transported to our laboratory immediately after purchase. Subsequently, each mussel was cleaned with natural seawater and maintained in some aquariums (6 L) with filtered natural seawater (18 ± 1 °C, 12 h light/12 h dark cycle). They were fed *Tetraselmis subcordiformis* (1 × 10^7^ cells/L). The natural seawater and algae were changed each day. After acclimation for at least 7 days, the individuals in good condition were selected for subsequent experiments.

The chlorophyte *T. subcordiformis* was purchased from the Institute of Aquatic Biology, Chinese Academy of Sciences. The DST-producing dinoflagellate, *P. lima* (CCMP 2579), which produced OA and DTX1 [52], was purchased from the National Center for Marine Algae and Microbiology (NCMA), and its fatty acid profiles are shown in Figure S2. The two algae were batch cultured in an artificial climate incubator (20 ± 1 °C, 60 µmol/ (m^2^ s), 12 h/12 h photoperiod), and f/2 silicon-free medium filtered through 0.22 µm filters.

### 4.2. Experimental Design

**Figure 7:**
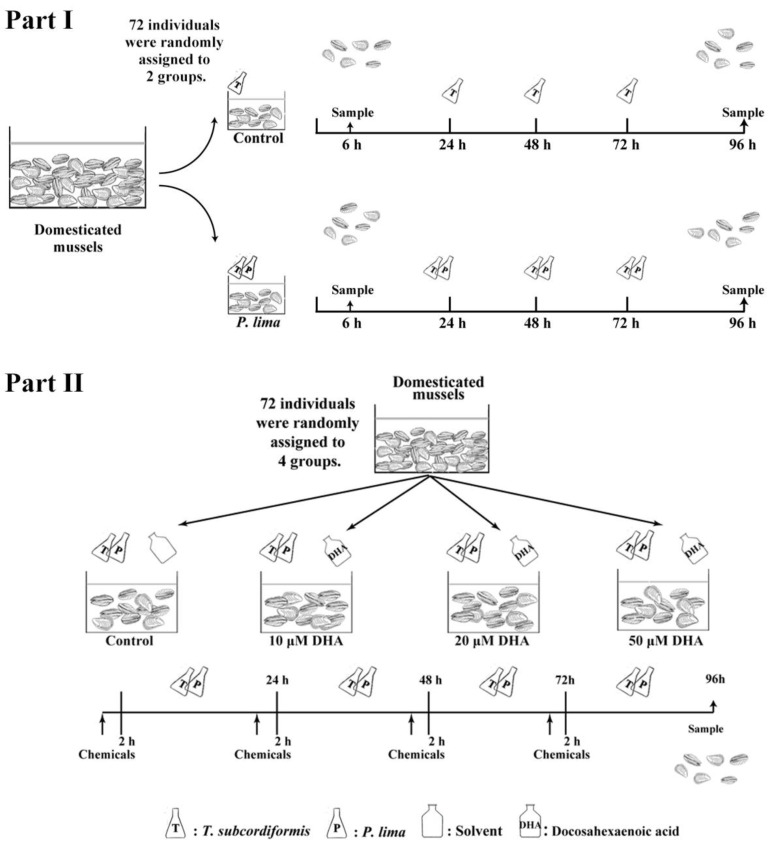
Experimental design. (**Part I**): Response of *P. viridis* to exposure of *P. lima*; (**Part II**): Effect of DHA in alleviating the toxicity of DSTs in *P. viridis*.

As DSTs primarily accumulate in the digestive gland, DST esterification, fatty acid levels, and antioxidant enzyme activities in the digestive gland tissues of the *P. lima*-exposed *P. viridis* with or without DHA were studied. The subsequent experiments were divided into two parts as shown in Figure 7. 

Part I: Response of *P. viridis* to *P. lima* exposure. In total, 72 *P. viridis* individuals were equally and randomly divided into two groups. The *P. lima*-exposed group was provided with *T. subcordiformis* (1 × 10^7^ cells/L) and *P. lima* (2 × 10^6^ cells/L), whereas the control group was provided only with *T. subcordiformis* (1 × 10^7^ cells/L). The seawater and microalgae were renewed regularly each day. The digestive gland tissues were collected after 6 h and 96 h of exposure. For minimizing errors due to individual differences, the tissues of six mussel individuals from the same group were pooled as one sample. Finally, three biological replicates were considered at each sampling time point for DST detection, fatty acid extraction, RNA extraction, and enzyme activity assay. 

Part II: Effect of DHA in alleviating the toxicity of DSTs in *P. viridis.* In total, 72 *P. viridis* individuals were equally and randomly divided into four groups: three DAH-added groups and one control. In the DHA-added groups, DHA (>99 % purity, MedChemExpress, Monmouth Junction, NJ, USA) was dissolved in dimethyl-sulfoxide (DMSO) and added to the tanks to different final concentrations (10, 20, and 50 µM). The control group was administered equal volume of DMSO (0.008% final concentration). After DHA or DMSO supplementation for 2 h, all the mussels were provided *T. subcordiformis* (1 × 10^7^ cells/L) and *P. lima* (2 × 10^6^ cells/L). The seawater was renewed daily at the same time and DMSO, DHA, and new microalgae were added as mentioned above. All the digestive gland tissues were collected after 96 h of exposure to *P. lima*. For minimizing individual differences, the tissues of six mussel individuals from the same group were pooled as one sample. Each group contained three biological replicates.

### 4.3. Toxin Analysis

Extraction and detection of DSTs were performed as described previously [39]. Briefly, a 1.0 g wet sample was homogenized with 3 mL of methanol (HPLC grade). After repeated extraction, the collected supernatant (10 mL) was dried under nitrogen and subsequently dissolved in 1 mL methanol for analyzing the free form of DSTs. Regarding esterified DSTs, 0.5 mL of methanolic extract was subjected to alkaline hydrolysis using 0.1 mL of 1.25 M sodium hydroxide at 76 °C for 40 min, followed by the addition of 1.25 M of hydrochloric acid (0.1 mL) for neutralization. The concentrations of the esterified DSTs were calculated by subtracting the value of the free-form DSTs from that of the total DSTs.

Liquid chromatography coupled to tandem mass spectrometry (LC-MS/MS, AB Sciex, Foster city, CA, USA) was performed on an AB Sciex equipped with a Turbospray ionization source QTRAP 4500 mass spectrometer (MS; AB Sciex Pte. Ltd., Singapore city, Singapore). A Poroshell 120 EC-C18 column (4.6 × 50 mm, 2.7 µm, Agilent Technologies, Wilmington, DE, USA) was used for separation of OA and DTX1 using a ternary mobile phase consisting of a 0.2% formic acid solution in water (solvent A), 100% acetonitrile (solvent B), and 10 mM ammonium acetate solution in water (solvent C). Multi-reaction monitoring mode (MRM) for negative ionization was used to detect OA and DTX1. As described in our previous study [42], the standards of OA (LC Laboratories, Woburn, MA, USA) and DTX1 (NRC, CAN), were diluted, and standard curves of chromatographic peak area (y) for OA and DTX1 against the toxin concentration (x) were established.

### 4.4. Analysis of Fatty Acids

Lipids were extracted from pre-lyophilized samples, and fatty acid composition was analyzed as fatty acid methyl esters using gas chromatography–mass spectrometry (GC-MS) [53]. N-nonadecyl ester (10 mg/mL) (purity ≥ 98, purchased from Macklin Biochemical Co., Ltd., Shanghai, China) was added as the internal standard. GC-MS/MS analysis was performed using an Agilent Technologies 7000C GC-MS with a HP-5MS column (30 m × 0.25 mm, 0.25 µm, Agilent Technologies). The column temperature was initially maintained at 65 °C for 3 min, and then increased at 10 °C/min to 165 °C, at 1 °C/min to 180 °C, at 15 °C/min to 270 °C, and at 285 °C for 3 min. The MS was performed in electron impact mode with a scan range of m/z 50–500 for the pseudo molecular ions at 70 eV.

### 4.5. RNA Extraction and Reverse Transcription-Quantitative Polymerase Chain Reaction (RT-qPCR)

The total RNA was extracted using the total RNA kit I (50) (R6934-01, Omega, Norcross, GA, USA). A NanoDrop 2000/2000c spectrophotometer was used to evaluate the concentration of RNA (Implen, Munich, Germany). HiScript^®^ II Q RT SuperMix for qPCR (+gDNA wiper) (R223-01, Vazyme, Nanjing, China) was used to reverse transcribe the cDNA from 1 µg total RNA. The PCR reaction mixture (20 µL) contained 0.2 µL of each primer, 2 µL of cDNA, 7.2 µL of ddH_2_O and 10 µL of AceQ^®^ qPCR SYBR^®^ Green Master Mix (Q111-03, Vazyme, Nanjing, China). PCR was performed using the CFX96 Real-Time PCR System (Bio-Rad, Hercules, CA, USA), and the PCR procedures were as follows: 95 °C for 30 s, 39 cycles of 95 °C for 10 s, and 60 °C for 30 s.

The genes encoding for ubiquitin A-52 (*Uba52*) and ribosomal protein L37 (*Rpl37*) were used as reference genes from among five genes, namely, those encoding elongation factor 1 alpha (*Ef1α*), ribosomal protein L3 (*Rpl3*), ribosomal protein L13-like (*Rpl13*), *Uba52,* and *Rpl37.* geNorm [54], NormFinder [55], and BestKepper [56] were used for analysis. The expression of *Nrf2*, *Keap1*, glutathione-S-transferases (*Gst*), glutathione reductase (*Gr*), superoxide dismutase (*Sod*), and glutathione peroxidase (*Gpx*) was evaluated using the normalized relative quantities (NRQ) formula [57]. All primers for qPCR were designed using Primer 6.0 (Table 1).

### 4.6. Detection of Oxidative Stress Biomarkers

The level of malondialdehyde (MDA) was measured using the lipid peroxidation MDA assay kit (Beyotime, Shanghai, China). SOD activity was measured using a commercial total SOD assay kit (NanJing JianCheng Bioengineering Institute, Nanjing, China). GR activity was measured using a commercial glutathione reductase (GR) assay kit (Beyotime) and GPx activity was detected using a commercial glutathione peroxidase (GPx) assay kit (Beyotime). The bicinchoninic acid protein assay kit (Beyotime) was used to determine protein content. All assays were performed according to the manufacturers’ instructions. A multi-mode microplate reader (Tecan Sunrise, Männedorf, Switzerland) was used to determine the absorbance in the experiments.

### 4.7. Histological Examination

Histological examination was performed as mentioned previously [52]. The digestive glands were carefully excised and immediately immersed in Boone’s fixative for at least 48 h. The paraffin-embedded tissues were sectioned at a 4 µm thickness using a manual rotary slicer (Leica RM2235, Lecia Microsystems Nussloch Gmbh, Heidelberger, Germany). After deparaffinization in xylene, rehydration using a gradient ethanol solution (100 % for 5 min, 90 % for 2 min, 70 % for 2 min), and subsequent staining with hematoxylin and eosin, the sections were sealed using neutral balsam. Finally, each stained slice was photographed using Pannoramic MIDI slide scanner (3DHISTECH, Budapest, Hungary) and analyzed using the Case-Viewer software (3DHISTECH, Budapest, Hungary). The scoring methods developed and modified by Bernet et al. (1999), Costa et al. (2013), and Joshyd et al. (2022) were used to assess the histopathological condition indices (HPI) in bivalves [49,58,59].

### 4.8. Statistical Analysis

All values were expressed as mean ± standard deviation (SD). Statistical analyses were performed using the SPSS Statistics 25.0 software (SPSS Inc., Chicago, IL, USA). The *t*-test and Welch’s t test were employed to determine the differences in mRNA level, fatty acid content, enzyme activity, and HPI between different groups. The difference in DSTs content between different groups was determined by one-way ANOVA followed by Duncan’s multiple range test with significant differences at *p* = 0.05.

## 5. Conclusions

In this study, we demonstrated the esterification of DSTs and found that the DHA content decreased significantly with DST esterification in the digestive gland of *P. viridis* after 96 h of exposure to *P. lima.* The addition of DHA significantly enhanced the esterification of DSTs and further increased the expression of Nrf2 signaling pathway-related genes and enzyme activity, ultimately alleviating the damage caused by DSTs. These results suggested that DHA may mitigate the toxic effects of DSTs by mediating the esterification of DSTs and activation of Nrf2 signaling pathway in *P. viridis.* Our findings provide new insights regarding the role of DHA against toxins in bivalves. However, how DHA activates the Nrf2 signaling pathway and promotes the esterification of DSTs in bivalves is still unclear and deserves further study.

## Figures and Tables

**Table 1:** Primer for qPCR.

Gene Name	Primer Sequence (5′-3′)		Amplicon Size (bp)
*Ef1α*	F:	CACTCCGTCTTCCACTCCA	131
	R:	CCTCTGGCATTGACTCGTG	
*Rpl3*	F:	GGTGGCACTATCTCCCAGAA	98
	R:	GCCATCTGGACGTTACACCT	
*Uba52*	F:	TTACATTTGGTCCTGCGTCTC	135
	R:	CAGTTGGTAGCCCTTTGATGA	
*Rpl13*	F:	TAAAGACTGGCAACGCTATGT	155
	R:	TCACAACTGGTCGGAGAAG	
*Rpl37*	F:	GTCGCAATAAGACGCACACGTTG	179
	R:	GTGCCTCATTCGACCAGTTCCG	
*Nrf2*	F:	TCAACCTGGACAGGAACCCA	90
	R:	TATCGCGACAGTGTGGACCT	
*Keap1*	F:	TATCGCTCCAATGAACACGG	173
	R:	AAGCACTTCTGGGGCTACGC	
*Gst*	F:	GTTGGCTCGAAATTAAGTATGGC	108
	R:	AAACTCCTCCAGTATTTTCTGGTCT	
*Gr*	F:	TTACTCCAGTTGCCATAGCAGCAG	113
	R:	TGGATGTGAGAACACCACAGTAGC	
*Gpx*	F:	CAACGACCCCCAGATTCAGA	80
	R:	TCTAGAGTCGGTAGGAGCCAT	
*Sod*	F:	GCAACATTCCTTCAGCACCT	154
	R:	CCTTGTTCCAAAAGCCTAATTG	

*Ef1α*, Elongation factor 1 alpha; *Rpl3*, Ribosomal protein L3; *Rpl13*, Ribosomal protein L13-like; *Rpl37*, Ribosomal protein L37; *Uba52*, Ubiquitin A-52; *Nrf2*, Nuclear factor erythroid 2-related factor 2; *Keap1*, Kelch-like ECH-associated protein-1; *Gst*, Glutathione S-transferase; *Gpx*, Glutathione peroxidase; *Gr*, Glutathione reductase; *Sod*, Superoxide dismutase.

## Data Availability

The data in this study are available from the corresponding author upon request.

## Supplementary Materials

The following supporting information can be downloaded at: https://www.mdpi.com/article/10.3390/md21030155/s1, Figure S1: The morphology of *Perna viridis* in the experiment. Figure S2: Relative abundance (%) of fatty acids in *Prorocentrum lima* strain CCMP 2579.
