# Supplementary material for: Possible Role of Docosahexaenoic Acid in Response to Diarrhetic Shellfish Toxins in the Mussel Perna viridis
Source: Mar Drugs. 2023 Feb 25;21(3):155. doi: 10.3390/md21030155 (PMC10058962; doi:10.3390/md21030155)
Supplement: Supplementary file 1 [file marinedrugs-21-00155-s001.zip › marinedrugs-2231446-supplementary.pdf]

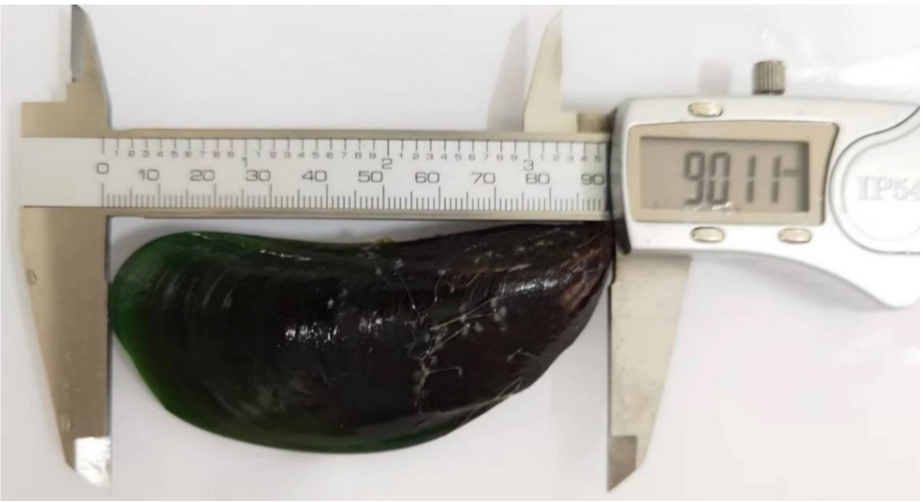

Supplementary Figure S1 The morphology of *Perna viridis* in the experiment.

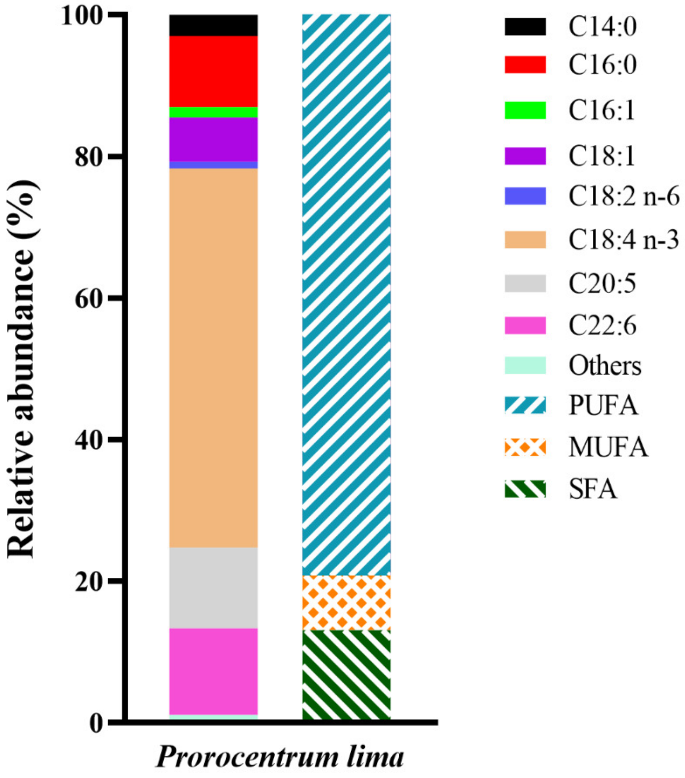

Supplementary Figure S2 Relative abundance (%) of fatty acids in *Prorocentrum lim*a strain CCMP 2579. SFA, MUFA and PUFA represent saturated fatty acids, monounsaturated fatty acids and polyunsaturated fatty acids, respectively.
